# Carbon-supported Ni nanoparticles for efficient CO_2_ electroreduction†
†Electronic supplementary information (ESI) available: Experimental details, TEM and HRTEM images, XPS survey, linear sweep voltammetry, cyclic voltammograms, Faradaic efficiency, and DFT calculation details. See DOI: 10.1039/c8sc03732a


**DOI:** 10.1039/c8sc03732a

**Published:** 2018-11-06

**Authors:** Mingwen Jia, Changhyeok Choi, Tai-Sing Wu, Chen Ma, Peng Kang, Hengcong Tao, Qun Fan, Song Hong, Shizhen Liu, Yun-Liang Soo, Yousung Jung, Jieshan Qiu, Zhenyu Sun

**Affiliations:** a State Key Laboratory of Organic–Inorganic Composites , College of Chemical Engineering , Beijing University of Chemical Technology , Beijing 100029 , P. R. China . Email: sunzy@mail.buct.edu.cn; b Graduate School of EEWS , Korea Advanced Institute of Science and Technology (KAIST) , Daejeon 34141 , Republic of Korea . Email: ysjn@kaist.ac.kr; c Department of Physics , National Tsing Hua University , Hsinchu , Taiwan 30013; d Technical Institute of Physics and Chemistry , Chinese Academy of Sciences , Beijing 100190 , P. R. China; e School of Chemical Engineering and Technology , Tianjin University , Tianjin 300072 , P. R. China

## Abstract

The development of highly selective, low cost, and energy-efficient electrocatalysts is crucial for CO_2_ electrocatalysis to mitigate energy shortages and to lower the global carbon footprint. Herein, we first report that carbon-coated Ni nanoparticles supported on N-doped carbon enable efficient electroreduction of CO_2_ to CO. In contrast to most previously reported Ni metal catalysts that resulted in severe hydrogen evolution during CO_2_ conversion, the Ni particle catalyst here presents an unprecedented CO faradaic efficiency of approximately 94% at an overpotential of 0.59 V, even comparable to that of the best single Ni sites. The catalyst also affords a high CO partial current density and a large CO turnover frequency, reaching 22.7 mA cm^–2^ and 697 h^–1^ at –1.1 V (*versus* the reversible hydrogen electrode), respectively. Experiments combined with density functional theory calculations showed that the carbon layer coated on Ni and N-dopants in carbon material both play important roles in improving catalytic activity for electrochemical CO_2_ reduction to CO by stabilizing *COOH without affecting the easy *CO desorption ability of the catalyst.

## Abstract 2

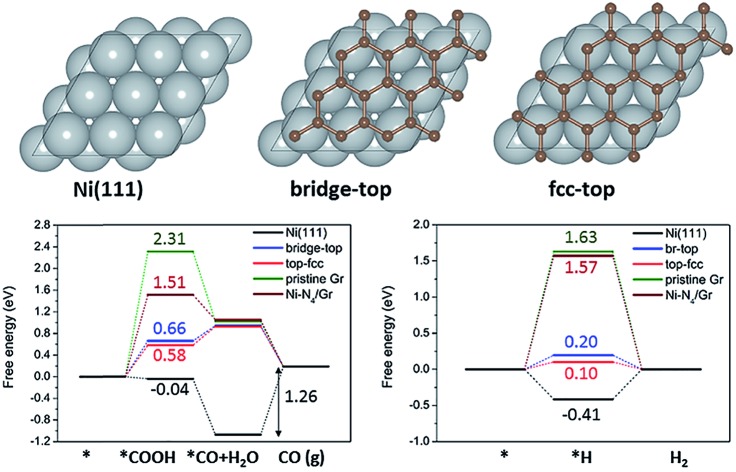
Carbon-coated Ni nanoparticles supported on N-doped carbon enable efficient electroreduction of CO_2_ to CO comparable to single Ni sites.

## Introduction

Direct electrochemical reduction of CO_2_ (ECR) powered by electricity from renewable sources provides a “clean” and efficient way to alleviate the greenhouse effect and to convert CO_2_ into value-added fuels and chemicals.^1^–^10^ Despite the recent progress made in ECR,^11^–^16^ it still suffers from (1) a large overpotential, (2) sluggish electron transfer kinetics, (3) insufficient product selectivity, and (4) degradation of catalytic activity within short periods. Additionally, proton reduction to generate H_2_ always takes place as a competitive reaction, especially in aqueous solutions, which lowers CO_2_ reduction selectivity and efficiency. Extensive efforts are therefore being devoted to developing new electrocatalysts that can reduce CO_2_ at high rates with low overpotentials and large turnover frequencies (TOFs).

The reduction of CO_2_ to CO [CO_2_ + 2H^+^ + 2e^–^ → CO + H_2_O, *E*0redox = –0.11 V *vs.* the reversible hydrogen electrode (RHE)] proceeds through a two-electron (e^–^)/proton (H^+^) transfer pathway.^7^ A CO_2_ molecule is first reduced to a carboxyl intermediate (*COOH) either by a concerted e^–^/H^+^ or by a decoupled e^–^ and H^+^ transfer that involves the formation of a CO_2_˙^–^ radical. Subsequently, a second e^–^/H^+^ attacks the oxygen atom (OH) in the *COOH to generate H_2_O (l) and CO. Au,^17^ Ag,^17^ modified Pd,^18^,^19^ and bimetallic Cu^20^ can tightly bind *COOH, which is further reduced to a *CO intermediate in aqueous media. The *CO is weakly bound to their surfaces, and CO desorbs from the metal electrodes as a major product. Despite their high CO_2_-to-CO conversion, the high cost and scarcity of these metals are problematic for practical applications. Exploration of cheap and earth-abundant catalysts for efficient CO_2_ electrocatalysis is thus desirable. Late transition metals such as Fe, Co, and Ni are promising alternatives to expensive noble metals. However, metallic Fe, Co, and Ni tend to promote the hydrogen evolution reaction (HER). They also possess strong bonding with adsorbed CO, dramatically limiting ECR. To solve this problem, construction of single metal sites has been demonstrated to enable engineering of the electronic properties of transition metals for enhanced ECR.^21^–^26^ It was speculated that charge transfer occurred between the metal atoms with delocalized electrons and the carbon 2p orbital in CO_2_ to form a CO_2_^δ–^ species, thereby reducing the energy barrier for ECR. Despite this success, there are still some unresolved issues associated with single atom electrocatalysis of CO_2_ reduction: (1) large-scale preparation of isolated single metal sites in a controlled manner remains a grand challenge, and (2) the mechanistic understanding of single-atom catalysis in ECR is elusive. Note that while most previous studies have focused on the correlation of adsorption and activation of CO_2_ with coordinatively unsaturated metal single atoms, the roles of coexistent metal particles and support surface species (such as nitrogen configurations) as well as possible metal particle–support interactions in ECR remain less explored. In this context, a question arises as to whether transition metal particles rather than single atoms can suppress the unwanted HER and afford efficient ECR by modulating the surface structure of metallic particles and the substrate surface groups.

To this end, we prepared carbon supported Ni nanoparticles (NPs) by pyrolysis of nickel diamine–dicarboxylic acid metal organic frameworks (MOFs) on carbon in argon. The synthetic procedure employed here is very simple and scalable by using a well-defined pyrolysis precursor. The properties of Ni were intentionally tailored by (1) coating it with a carbon layer and (2) tuning the support surface species by manipulation of the MOF linker type. Interestingly, we found that modification of the Ni NPs by combining the two strategies can significantly enhance the electrochemical reduction of aqueous CO_2_ to CO, providing a high CO faradaic efficiency (FE), large CO partial current density, and remarkable formation TOF. To the best of our knowledge, this is the first report on the capability of Ni NPs for catalyzing CO_2_ electroreduction with high efficiency. Furthermore, density functional theory (DFT) calculations revealed that Ni can play an important role in stabilizing *COOH and improving catalytic activity on the carbon coating on Ni surfaces. Also, pyrrolic N and graphitic N sites in the N-doped carbon support enhance the catalytic activity of pristine carbon.

## Results and discussion

Typically, N-doped carbon supported Ni nanoparticles (denoted as Ni-NC_X@C) were synthesized first by reaction of nickel(ii) nitrate hexahydrate with 1,4-diazabicyclo[2.2.2]octane (DABCO) and an organic linker *X* [*X* = terephthalic acid (TPA), 2-aminoterephthalic acid (ATPA), 2,5-dichloroterephthalic acid (DCTPA), 2,5-dibromoterephthalic acid (DBTPA), or 2,3,4,6-tetrachloroterephthalic acid (TCTPA)] in the presence of carbon black in dimethylformamide (DMF) at 150 °C, followed by annealing at 800 °C for 2 h under an Ar atmosphere (for details, see the ESI†). A number of different Ni catalysts were obtained by changing the organic linker type to tune support surface species. The content of Ni in the catalysts was determined by inductively coupled plasma-atomic emission spectrometry (ICP-AES); it was ∼3.6 wt% in Ni-NC_ATPA@C and ∼4.0 wt% in Ni-NC_TPA@C.

**Fig. 1:**
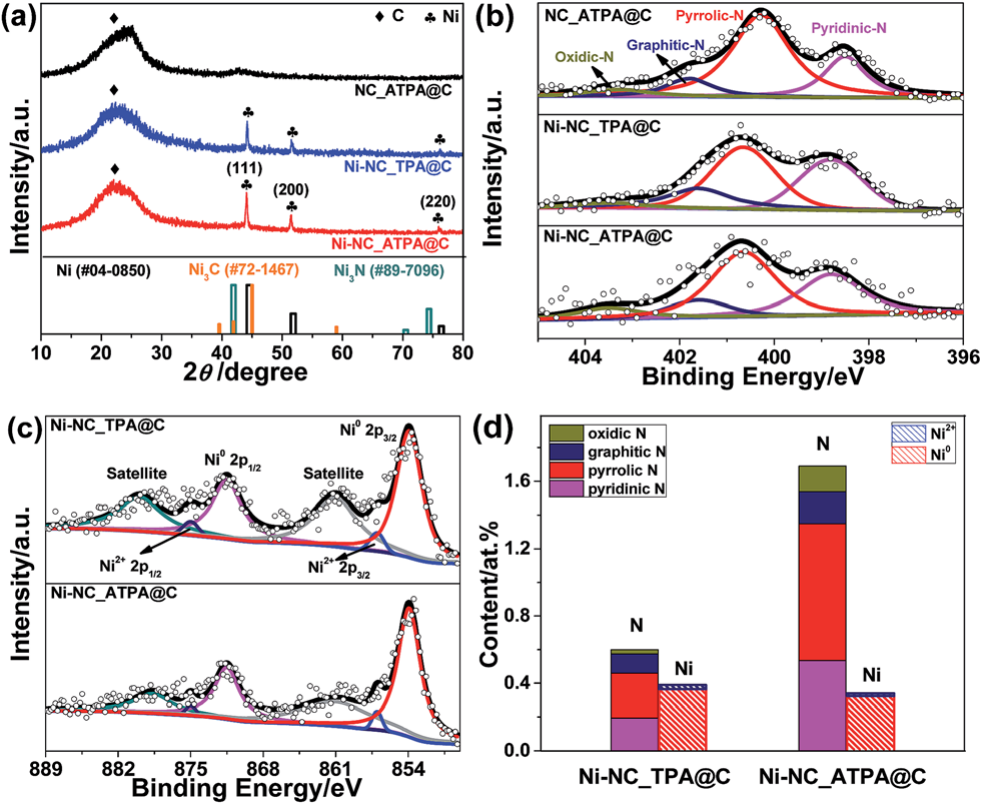
(a) XRD patterns and (b) N 1s XPS spectra of N doped carbon without Ni (NC_ATPA@C), Ni-NC_TPA@C, and Ni-NC_ATPA@C. (c) Ni 2p XPS spectra. (d) Atomic contents of N species including pyridinic N, pyrrolic N, graphitic N, and oxidic N, and Ni species including Ni^0^ and Ni^2+^ of Ni-NC_TPA@C and Ni-NC_ATPA@C based on XPS.

The X-ray diffraction (XRD) patterns of Ni-NC_TPA@C and Ni-NC_ATPA@C, as shown in Fig. 1a, both present three sharp diffraction peaks at approximately 44.5°, 51.8°, and 76.4°, corresponding well with the (111), (200) and (220) planes of Ni^0^ respectively (PDF #04-0850), in addition to a broad peak at about 23° originating from the (002) reflection of the graphitic structure. No reflection peaks of nickel nitride (PDF #89-7096) and nickel carbide (PDF #72-1467) were identified, suggesting the predominant formation of metallic Ni in the samples. The average crystallite size was determined to be about 34.6 nm for Ni-NC_ATPA@C and 31.0 nm for Ni-NC_TPA@C from the (111) reflection by utilizing Scherrer's equation relating the coherently scattering domains with Bragg peak widths: *L* = *kλ*/*B* cos(*θ*), in which *k* = 0.89 for spherical particles and *B* is the full angular width at half-maximum of the peak in radians.

The catalyst surface chemical composition and state were studied by X-ray photoelectron spectroscopy (XPS) (Fig. S1†). The high-resolution N 1s XPS spectra were deconvoluted into pyridinic N (binding energy, BE: ∼398.6 eV), pyrrolic N (BE: ∼400.7 eV), graphitic (quaternary) N (BE: ∼401.6 eV), and oxidic N (BE: ∼403.5 eV) in all cases.^27^ The typical N–Ni peak centered at ∼399.6 eV^28^ was not observed for Ni-NC_TPA@C or Ni-NC_ATPA@C (Fig. 1b). The position of the pyrrolic N peak shifted to a higher BE value for the Ni catalysts compared to the sample without Ni, which is probably due to charge transfer between Ni and pyrrolic N species. While no N 1s XPS peak at 397 eV typical of nickel nitride was identified, ruling out the formation of nitrides, in both Ni-NC_TPA@C and Ni-NC_ATPA@C, Ni 2p_3/2_ is dominated by a peak at ∼853.8 eV, which can be assigned to metallic Ni^0^ (Fig. 1c). The other possible minor peak at 857.1 eV is ascribed to Ni^2+^, resulting from Ni^0^ oxidation upon exposure to air. However, the peak at ∼856.0 eV corresponding to low-valent Ni(i) bound to N was not found, excluding the formation of Ni single atoms in both catalysts. Based on XPS, the Ni-NC_ATPA@C is estimated to have a slightly smaller loading of Ni but remarkably higher N content (∼1.7 at% overall N, ∼0.81 at% pyrrolic N, ∼0.53 at% pyridinic N, and ∼0.19 at% graphitic N species) than the Ni-NC_TPA@C (∼0.6 at% overall N, ∼0.26 at% pyrrolic N, ∼0.20 at% pyridinic N, and ∼0.11 at% graphitic N species) (Fig. 1d). Both XRD and XPS analyses confirmed the major form of metallic Ni rather than single Ni atoms in the resulting Ni catalysts.

**Fig. 2:**
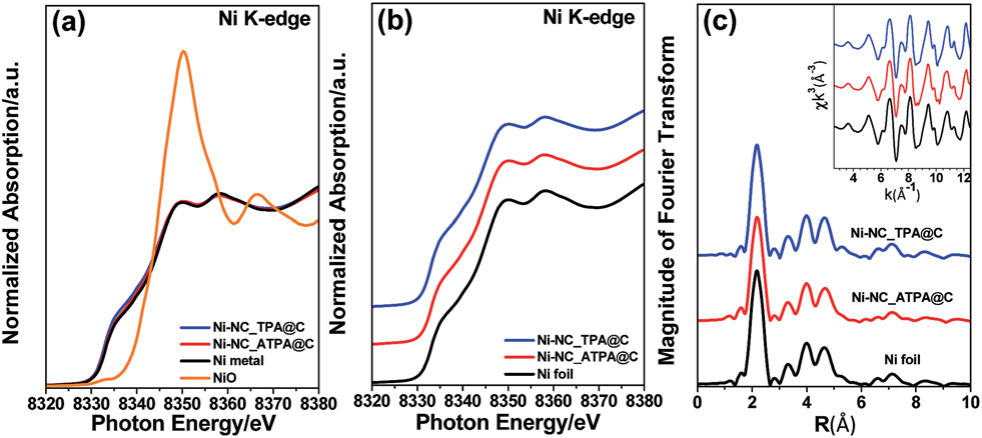
(a) Ni K-edge X-ray absorption fine structure spectra of Ni-NC_TPA@C and Ni-NC_ATPA@C along with those of NiO and Ni foil as reference standards. (b) Enlarged XANES spectra and (c) Fourier transformed EXAFS spectra of the Ni K-edge for Ni-NC_TPA@C, Ni-NC_ATPA@C, and Ni foil.

Fig. 2a shows the X-ray absorption near edge structure (XANES) of Ni-NC_TPA@C and Ni-NC_ATPA@C as well as nickel foil and nickel oxide for comparison. The Ni absorption edge positions of Ni-NC_TPA@C and Ni-NC_ATPA@C are both consistent with that of the Ni metal standard (Fig. 2b). No Ni(i) or Ni(ii) moieties were detectable, indicating that Ni was present in the metallic state rather than in the form of single Ni sites. Fourier transformed extended X-ray absorption fine structure (EXAFS) analysis also confirmed the predominant presence of Ni–Ni bonds and the absence of Ni–N bonds in the pyrolysis products, in agreement with the XRD and XPS results (Fig. 1a–c).

**Fig. 3:**
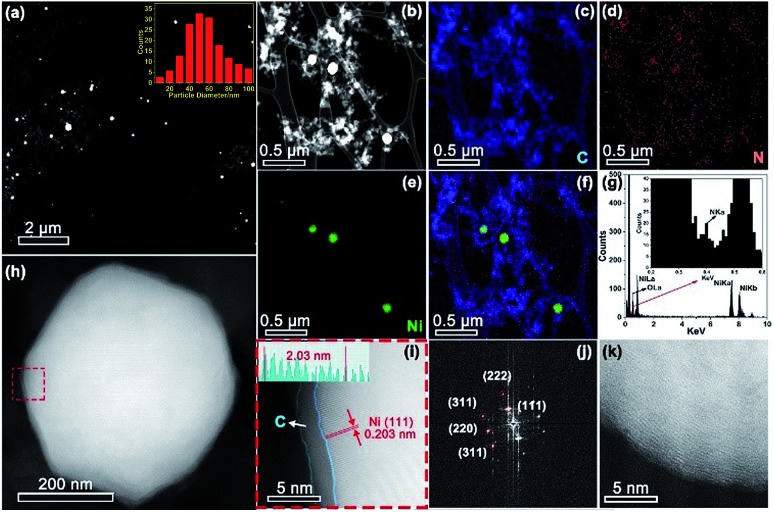
(a and b) STEM images of Ni-NC_ATPA@C. Inset in (a): size distribution of Ni NPs. EDX maps of (c) C, (d) N and (e) Ni. (f) Overlay EDX map. (g) EDX spectrum of the region shown in (b). (h) STEM image of an individual Ni NP. (i) Enlarged STEM image of the area enclosed in the red dashed square in (h), manifesting an outer carbon layer around the crystalline Ni NP. (j) Fast Fourier transform (FFT) of the region shown in (i). (k) STEM image of the carbon support.

Aberration corrected high-angle annular dark-field scanning transmission electron microscopy (HAADF-STEM) observation (Fig. 3a and b) revealed the formation of Ni nanoparticles, with diameters ranging from 10 to 100 nm (inset of Fig. 3a). The brighter spots in the STEM images can be assigned to Ni particles because of the higher atomic number of Ni than C. The energy-dispersive X-ray spectroscopy (EDX) maps (Fig. 3c–f) along with the EDX spectrum (Fig. 3g) confirmed the presence of Ni within the N doped carbon matrix. However, no single Ni atoms or subnanometer Ni structures, which should appear as bright dots in HAADF images if present, were detected even after careful examination of different regions by HAADF-STEM. The sample exhibited strong ferromagnetic properties (Fig. S2†), providing additional evidence that single Ni atoms unlikely predominated. Combining the XRD, XPS, XANES, EXAFS, and HAADF-STEM results, we infer that Ni exists mainly in metallic nanoparticles instead of single sites. Most of the crystalline Ni particles were observed to be wrapped by carbon shells (Fig. 3h–j) and deposited on a graphitic support (Fig. 3k). This carbon shell likely prevents the Ni NPs from coming into direct contact with the aqueous electrolyte, thereby significantly suppressing the HER. The role of this shell in ECR will be discussed further in the following DFT part. A similar morphology has also been observed for Ni-NC_TPA@C.

**Fig. 4:**
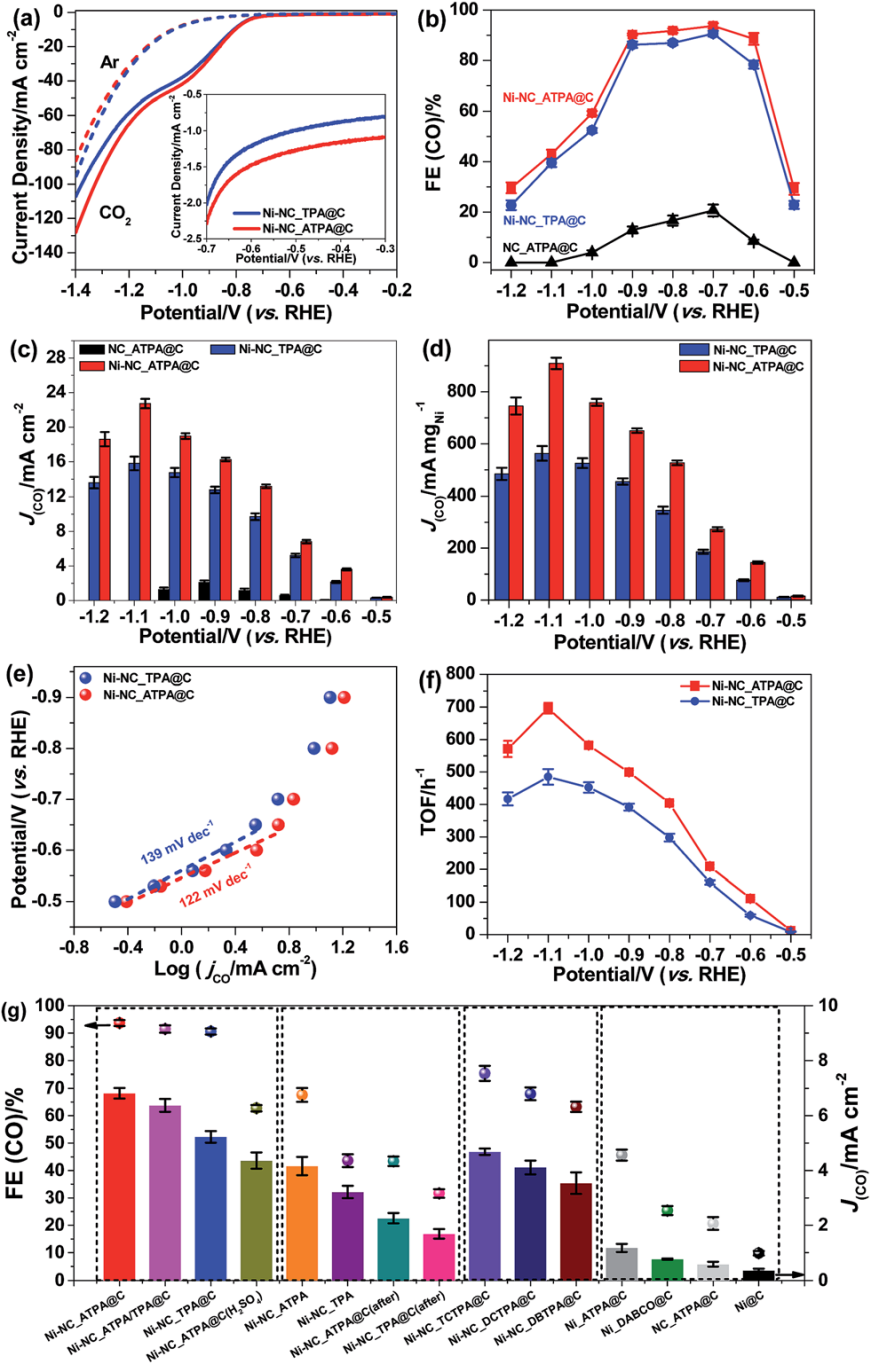
(a) The LSV results of Ni-NC_TPA@C and Ni-NC_ATPA@C on a glassy carbon electrode in Ar- (dashed line) or CO_2_ (solid line)-saturated 0.5 M KHCO_3_ with a scan rate of 5 mV s^–1^. The inset highlights the LSV curves in the potential range from –0.3 to –0.7 V. (b) The CO FEs and (c) partial current densities of Ni-NC_TPA@C, Ni-NC_ATPA@C, and NC_ATPA@C at various applied potentials. (d) Mass activities, (e) Tafel plots, and (f) TOFs for CO production of Ni-NC_TPA@C and Ni-NC_ATPA@C at different potentials. (g) The CO FEs and partial current densities at –0.7 V over Ni-NC_ATPA@C, Ni-NC_ATPA/TPA@C (using a mixture of TPA and ATPA as a linker with a molar ratio of 1 : 1), Ni-NC_TPA@C, Ni-NC_ATPA@C after H_2_SO_4_ washing; Ni-NC_ATPA and Ni-NC_TPA without adding carbon black during synthesis, Ni-NC_ATPA@C (after) and Ni-NC_TPA@C (after) with carbon black added afterwards; Ni-NC_TCTPA@C, Ni-NC_DCTPA@C, and Ni-NC_DBTPA@C prepared by using a halogen-based compound linker; Ni_ATPA@C, Ni_DABCO@C, NC_ATPA@C and Ni@C electrodes.

The carbon supported Ni nanoparticle catalysts were subjected to electrochemical CO_2_ reduction tests in a gas-tight three-electrode configuration cell. All potentials reported in this work are with respect to the RHE scale unless specified otherwise. Shown in Fig. 4a are the linear sweep voltammetry (LSV) results of Ni-NC_TPA@C and Ni-NC_ATPA@C in 0.5 M aqueous KHCO_3_ saturated with Ar or CO_2_. A cathodic peak at about –0.6 V was observed in the LSV curves. The gaseous and liquid products were analyzed by gas chromatography (GC) and ^1^H nuclear magnetic resonance (NMR). Only CO and/or H_2_ were detectable, but no liquid products were detected under applied potentials in the range of –0.5 to –1.2 V in a CO_2_-saturated 0.5 M KHCO_3_ solution (pH 7.2) at room temperature and atmospheric pressure. As shown in Fig. 4b, CO_2_-to-CO conversion starts from potentials ≥–0.5 V and increases to a maximum CO FE at –0.7 V. The CO FE follows the trend of Ni-NC_ATPA@C > Ni-NC_TPA@C, which likely correlates with the trend of the content of overall N and pyrrolic N species. Both Ni samples dramatically outperform the N-doped carbon catalyst without metal (Fig. 4b and S3†). Notably, the maximum CO FE is up to 93.7% for the Ni-NC_ATPA@C, making it superior to previously reported carbon supported Ni NPs (maximum CO FE < 25%) and Ni single atoms derived from ZIF-8 (maximum CO FE = 71.9% at –0.9 V),^12^ and even comparable to the best single Ni site catalysts (Table S1†).^24^,^25^,^29^–^31^ The competitive HER was substantially inhibited with H_2_ FE less than 10.0%, in sharp contrast to the severe production of H_2_ (H_2_ FE ≥ 90%) over supported Ni nanoparticle catalysts as reported in the literature.^30^ Both CO partial current density and mass activity increased as a function of overpotential with Ni-NC_ATPA@C outperforming Ni-NC_TPA@C (Fig. 4c and d), with the corresponding values above 13.2 mA cm^–2^ and 527 mA mg_Ni_^–1^, respectively for Ni-NC_ATPA@C at potentials of –0.8 to –1.2 V. The maximum values of the two parameters reached as high as 22.7 mA cm^–2^ and 910 mA mg_Ni_^–1^, respectively at –1.1 V.

The Tafel slope (Fig. 4e), an indication of the kinetics for CO formation, is ∼122 mV dec^–1^ for Ni-NC_ATPA@C, lower than 139 mV dec^–1^ for Ni-NC_TPA@C. This suggests that the formation of the *COOH intermediate on the surface of both catalysts determines the reaction rate. But Ni-NC_ATPA@C has faster kinetics for CO_2_ reduction than Ni-NC_TPA@C. Likewise, Ni-NC_ATPA@C exhibited larger CO formation TOFs than Ni-NC_TPA@C (Fig. 4f and S4†), and achieved 697 h^–1^ at –1.1 V based on electrochemical surface area determination from double layer capacitance measurements. The Ni-NC_ATPA@C catalyst retained a stable current density over 6 mA cm^–2^ and a CO FE of about 93% after electrolysis for 24 h at –0.7 V (Fig. S5†).

In order to probe the active centers in carbon supported Ni catalysts, control experiments were performed on different Ni catalysts that were produced with varying linkers, as displayed in Fig. 4g. It was found that N doped carbon in the absence of Ni (NC_ATPA@C) showed very low ECR activity toward CO generation (Fig. 4b, c, and g, and S3†), suggesting that Ni is responsible for efficient ECR. Nevertheless, Ni particles supported on carbon black without nitrogen modification (Ni@C) also exhibited poor ECR activity. This indicates that N doping plays an important role in facilitating CO_2_-to-CO conversion. The ECR performance in terms of both CO FE and partial current density increased with N content. This can be evidenced by the significantly lower activity of the two Ni catalysts prepared by using only ATPA (Ni_ATPA@C) or DABCO (Ni_DABCO@C) without addition of another organic linker, which have lower N content as compared with Ni-NC_TPA@C, Ni-NC_TPA/ATPA@C, and Ni-NC_ATPA@C. We also made efforts to modify the carbon support with N and halogen (Cl or Br) atoms by using DCTPA, DBTPA, or TCTPA as a linker. However, the resultant Ni catalysts supported on N, Cl or Br co-doped carbon did not show improved ECR activity compared with Ni-NC_TPA@C. The Ni-NC_TPA and Ni-NC_ATPA without incorporation of carbon black, and Ni-NC_TPA@C (after) and Ni-NC_ATPA@C (after) with equivalent amounts of carbon black mixed afterwards all have lower CO FEs and partial current densities relative to the corresponding Ni catalysts with addition of carbon black during the preparation process. The pyrolysis temperature was observed to considerably influence the ECR performance of the catalyst as well, with 800 °C likely being the optimal reaction temperature (Fig. S6†). Furthermore, we found that acid treatment of Ni_NC_ATPA@C in 2 M H_2_SO_4_ for 1 h led to a pronounced decrease of the CO FE from ∼93.7% to ∼62.7%, highlighting that Ni NPs contribute to the ECR (Fig. 4g).

**Fig. 5:**
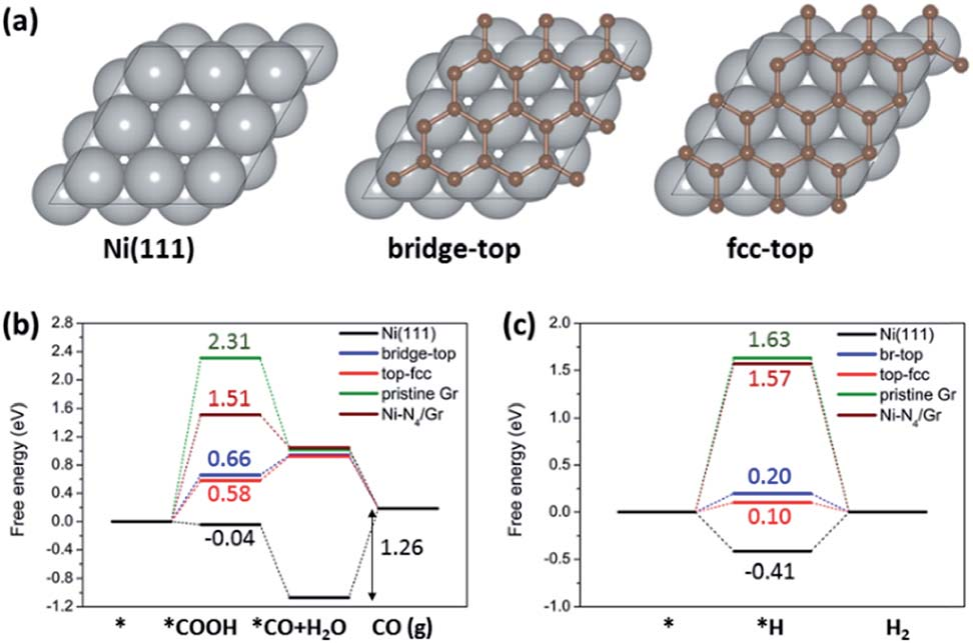
(a) Calculation models for Ni(111) and two graphene structures (bridge-top and fcc-top) for Gr/Ni(111). The free energy diagrams for (b) electrochemical CO_2_ reduction to CO and (c) the HER on Ni(111), Gr/Ni(111), pristine graphene and Ni–N_4_/Gr.

To investigate the effects of carbon coating on Ni nanoparticles and nitrogen content in carbon supports on the catalytic activity and selectivity of ECR, we performed DFT calculations. We considered a graphene (Gr) monolayer on a Ni(111) surface (Gr/Ni(111)) to model the carbon coating on Ni nanoparticles. Previous studies on graphene on Ni(111) by using high-resolution X-ray photoelectron spectroscopy (HR-XPS) combined with DFT calculations showed that the two different graphene structures (bridge-top and top-fcc) have almost identical energies and both structures are experimentally detected on Ni simultaneously.^32^ We also found that the energy difference between these two structures is 0.003 eV per C atom; thus, we considered both structures as well in a carbon coated model on Ni (Fig. 5a). For comparison, we also considered Ni(111), pristine graphene, and Ni–N_4_ embedded graphene (Ni–N_4_/Gr), which has been reported as an efficient Ni catalyst for ECR.^24^,^25^ Based on the XPS results (Fig. 1b), graphitic, pyridinic, and pyrrolic N were considered for investigating the effect of nitrogen content in carbon supports. The optimized structures of reaction models and intermediates are shown in the ESI (Fig. S7–S9†).

The free energy diagrams of electrochemical CO_2_ reduction to CO (Fig. 5b) indicate improved catalytic activity of Gr/Ni(111) compared to the other catalysts under comparison here. Pristine graphene shows a high reaction free energy for *COOH formation (2.31 eV), which is the first protonation step for electrochemical CO_2_ reduction to CO. Interestingly, Gr/Ni(111) displays a significantly lowered free energy change for *COOH formation (0.58–0.66 eV), even lower than that of Ni–N_4_/Gr (1.51 eV) reported previously as an efficient catalyst for the same reaction. Ni(111) shows a lower free energy change for *COOH formation. However, Ni(111) requires a large energy penalty for CO desorption (1.26 eV), indicating overall difficult CO production on the bulk Ni(111) surfaces. In contrast to Ni(111), CO desorption is exothermic on Gr/Ni(111), pristine graphene, and Ni–N_4_/Gr. However, it is important to emphasize that, in Gr/Ni(111), Ni(111) plays an important role in improving catalytic activity by greatly stabilizing the *COOH adsorption on graphene but not affecting the easy CO desorption ability of graphene.

In the presence of N in the carbon support, the free energy change for *COOH formation increases in the order of pyrrolic N (–0.71 eV) < pyridinic N (–0.06 eV) < graphitic N (1.34 eV) (Fig. S10†). All of these reaction free energies are lower than that of pristine graphene (2.31 eV), indicating that N can facilitate stabilization of *COOH compared to pristine graphene. The free energy change for CO desorption is also lowered on pyrrolic N (0.79 eV) and graphitic N (–0.79 eV) compared to that of Ni(111) (1.26 eV), except for pyridinic N (1.69 eV). These results suggest that N-doping, particularly in the form of pyrrolic N and graphitic N, can indeed prompt electrochemical CO_2_ reduction to CO on the carbon support material, consistent with experiments.

We also found that the hydrogen evolution reaction (HER), which is the most problematic yet dominant side reaction in ECR, can be suppressed on Gr/Ni(111) compared to Ni(111) (Fig. 5c). For the HER, we focused on the strength of H adsorbed on the surface (*H) since that determines the thermodynamic feasibility of the HER and, more importantly, acts as an active site to block ECR.^33^ The free energy change for *H formation is highly negative on Ni(111) (–0.41 eV), indicating that *H covers the Ni(111) surface very easily. On the other hand, on Gr/Ni(111), the free energy change for *H formation is much less favorable (0.10–0.20 eV), meaning that Gr/Ni(111) will be less covered by *H with the active sites made available for ECR.

## Conclusions

In summary, we demonstrate that modification of metallic Ni by a combination of carbon coating and incorporation of a N-doped carbon support can effectively suppress the HER and significantly enhance the electroreduction of aqueous CO_2_ to CO. This metallic Ni catalyst affords a high CO FE of up to approximately 94% (at –0.7 V *vs.* RHE) and a current density of 22.7 mA cm^–2^ (at –1.1 V), making it superior to all previous metallic Ni catalysts and even comparable to the best single Ni atom catalysts reported to date. Manipulation of the organic linker type and the addition of carbon black enable tuning of the catalytic properties of the resulting Ni nanoparticles. DFT calculations demonstrate that Ni greatly stabilizes the adsorption of *COOH on Gr/Ni(111) (increased activity) compared to pristine graphene without compromising the easy *CO desorption ability of pristine graphene (selectivity). Also, adding nitrogen-dopants (mainly graphitic N and pyrrolic N configurations) to the carbon support is shown to play an important role in improving catalytic activity by stabilizing *COOH. We believe that this work provides a new scheme to design low cost and active CO_2_ reduction catalysts with high selectivity toward CO, which is of importance to both fundamental mechanism studies and technological applications in ECR.

## Conflicts of interest

The authors of this manuscript have no conflicts of interest.

## Author contributions

M. J., C. M. and H. T. prepared the samples and conducted electrocatalytic tests. M. J., Q. F. and S. L. performed XRD, XPS, FTIR, and SEM characterization experiments. C. C. and Y. J. performed DFT calculations. S. H. performed STEM measurements. T. W. and Y. S. performed EXAFS tests. P. K. and J. Q. helped with discussions on the electrocatalytic results. Z. S. supervised the project and wrote the manuscript. All authors discussed the results and commented on the manuscript.

## Supplementary Material

Supplementary information
