# Supplementary material for: Carbon-supported Ni nanoparticles for efficient CO2 electroreduction
Source: Chem Sci. 2018 Nov 6;9(47):8775–80. doi: 10.1039/c8sc03732a (PMC6335639; doi:10.1039/c8sc03732a)
Supplement: Supplementary file 1 [file SC-009-C8SC03732A-s001.pdf]

Electronic Supplementary Material (ESI) for Chemical Science.
This journal is © The Royal Society of Chemistry 2018

Supplementary Information

Carbon-supported Ni nanoparticles for efficient CO_2 Electroreduction

Mingwen Jia[a], Changhyeok Choi[b], Tai-Sing Wu[c], Chen Ma[d], Peng Kang[d,e], Hengcong Tao[a], Qun Fan[a], Song Hong[a], Shizhen Liu[a], Yun-Liang Soo[c], Yousung Jung[b,*], Jieshan Qiu[a] and Zhenyu Sun [a,*]

[a] *State Key Laboratory of Organic-Inorganic Composites, College of Chemical Engineering, Beijing University of Chemical Technology, Beijing 100029, P.R. China. E-mail: sunzy@mail.buct.edu.cn*

[b] *Graduate School of EEWS, Korea Advanced Institute of Science and Technology (KAIST), Daejeon 34141, Republic of Korea. E-mail: ysjn@kaist.ac.kr*

[c] *Department of Physics, National Tsing Hua University, Hsinchu, Taiwan 30013*

[d] *Key Laboratory of Photochemical Conversion and Optoelectronic Materials Technical Institute of Physics and Chemistry, Chinese Academy of Sciences, Beijing 100190, P.R. China*

[e] *School of Chemical Engineering and Technology, Tianjin University, Tianjin 300072, P.R. China*

Synthesis of Ni-NC_APTA@C and other catalysts. Nickel(II) nitrate hexahydrate (44.59 mg) and 1,4-diazabicyclo[2.2.2]octane (DABCO; 76.41 mg) were added into 1.5 mL of *N*, *N*-dimethylformamide (DMF) in a round bottom at room temperature and then the mixture was stirred for 2 min. Then, 2-aminoterephthalic acid (ATPA; 83.32 mg) dissolved in 1.0 mL thermal DMF was added into the mixture, which was subjected to heating by refluxing in an oil bath at 150 ºC for 30 min. Subsequently, 150 mg of carbon powder (Vulcan XC72R) with 1.5 mL DMF was added into the reaction mixture by heating at 150 ºC for another 5 h. Afterwards, the DMF in the reaction products was evaporated at 150 ºC for 20 h to grow Ni-MOFs on carbon. The completely dried products were grinded to powder and pyrolyzed at 800 ºC for 2 h under an argon atmosphere.

The same procedure has been applied for the preparation of

(1) Ni-NC_ATPA/TPA@C (44.59 mg nickel (II) nitrate hexahydrate, 51.6 mg 1,4-diazabicyclo[2.2.2]octane, 38.21 mg terephthalic acid, 41.66 mg 2-aminoterephthalic acid and 150 mg Vulcan XC72R),

(2) Ni-NC_TPA@C (44.59 mg nickel (II) nitrate hexahydrate, 51.6 mg 1,4-diazabicyclo[2.2.2]octane, 76.41 mg terephthalic acid and 150 mg Vulcan XC72R),

(3) Ni-NC_ATPA@C (H_2SO_4) (soaking Ni-NC_APTA@C in 2 M H_2SO_4 for 1 h)

(4) Ni-NC_ATPA (44.59 mg nickel (II) nitrate hexahydrate, 51.6 mg 1,4-diazabicyclo[2.2.2]octane and 83.32 mg 2-aminoterephthalic acid),

(5) Ni-NC_TPA (44.59 mg nickel (II) nitrate hexahydrate, 51.6 mg 1,4-diazabicyclo[2.2.2]octane and 76.41 mg terephthalic acid),

(6) Ni-NC_ATPA@C (after) (mix Ni-NC_ATPA and Vulcan XC72R by 2 to 3),

(7) Ni-NC_TPA@C (after) (mix Ni-NC_TPA and Vulcan XC72R by 2 to 3),

(8) Ni-NC_TCTPA@C (44.59 mg nickel (II) nitrate hexahydrate, 51.6 mg 1,4-diazabicyclo[2.2.2]octane, 139.6 mg tetrachloroterephthalic acid and 150 mg Vulcan XC72R),

(9) Ni-NC_DCTPA@C (44.59 mg nickel (II) nitrate hexahydrate, 51.6 mg 1,4-diazabicyclo[2.2.2]octane, 108.01 mg 2,5-dichloroterephthalic acid and 150 mg Vulcan XC72R),

(10) Ni-NC_DBTPA@C (44.59 mg nickel (II) nitrate hexahydrate, 51.6 mg 1,4-diazabicyclo[2.2.2]octane, 148.98 mg 2,5-dibromoterephthalic acid and 150 mg Vulcan XC72R),

(11) Ni_ATPA@C (44.59 mg nickel (II) nitrate hexahydrate, 83.32 mg 2-aminoterephthalic acid and 150 mg Vulcan XC72R),

(12) Ni-DABCO@C (44.59 mg nickel (II) nitrate hexahydrate, 51.6 mg 1,4-diazabicyclo[2.2.2]octane and 150 mg Vulcan XC72R)

(13) Ni@C (44.59 mg nickel (II) nitrate hexahydrate and 150 mg Vulcan XC72R).

(14) NC_ATPA (76.41 mg 1,4-diazabicyclo[2.2.2]octane, 83.32 mg 2-aminoterephthalic acid and 150 mg Vulcan XC72R).

Characterization. XPS experiments were carried out using Thermo Scientific ESCALAB 250Xi instrument. The instrument was equipped with an electron flood and scanning ion gun. All spectra were calibrated to the C 1s binding energy at 284.8 eV. X-ray powder diffraction (XRD) was performed with a D/MAX-RC diffractometer operated at 30 kV and 100 mA with Cu Kα radiation. High-angle annular dark field scanning TEM (HAADF-STEM) was conducted using a JEOL ARM200 microscope with 200 kV accelerating voltage. STEM samples were prepared by depositing a droplet of suspension onto a Cu grid coated with a lacey carbon film. The XAFS measurements were performed in fluorescence mode using a Lytel detector at beam line BL07A of Taiwan Light Source, NSRRC. A Si(111) Double Crystal Monochromator (DCM) was used to scan the photon energy. The energy resolution ($\Delta E/E$) for the incident X-ray photons was estimated to be 2×10^{-4}. Quantitative information on the radial distribution of neighboring atoms surrounding Ni was derived from the extended absorption fine structure (EXAFS) data. An established data reduction method was used to extract the EXAFS χ-functions from the raw experimental data using the IFEFFIT software. To ascertain the reproducibility of the experimental data, at least two scans were collected and compared for each sample.

Electrochemical measurements. Electrochemical measurements were evaluated in a gas-tight three electrode configuration cell using aqueous $KHCO_3$ electrolyte, with Pt wire as the counter electrode, and saturated Calomel reference electrode (SCE) as the reference electrode. The working electrodes were fabricated by dropping sample suspension onto the pre-polished glassy carbon electrodes. Briefly, 10 mg of a catalyst powder was dispersed into 1 mL of ethanol with 10 μL of 0.5 wt% Nafion solution. The mixture was ultrasonicated for 45 min to form a uniform suspension. Then 10 μL of dispersed catalyst ink was dropped onto the glassy carbon electrode (3 mm in diameter). The linear sweep voltammetry (LSV) and controlled potential

electrolysis measurements were recorded in Ar- or CO_2-saturated 0.5 M $KHCO_3$ solution. The potentials were controlled by an electrochemical working station (CHI 601E, Shanghai CH Instruments Co., China). All potentials in this study were measured against the SCE and converted to the RHE reference scale using the equation:

E (*vs*. RHE) = E (*vs*. SCE) + 0.0245 + 0.0591 × pH (Eq. 1)

The gas-phase product analysis for the electrochemical experiments was carried out using an SRI 8601C MG#1 gas chromatograph (GC) system. The liquid product was analyzed in DMSO-d6 with tetramethylsilane (TMS) as an internal standard by ^{1}H nuclear magnetic resonance (NMR) (Bruker Avance III 400 HD spectrometer). No liquid products including formate were detectable by ^{1}H NMR at -0.8 V (vs. RHE) in CO_2-saturated 0.5 M $KHCO_3$ solution. This suggests that CO and H_2 were the main products as detected by GC.

Number of active sites and turn over frequency (TOF) measurements. We applied a roughness factor technique to determine the number of active edge sites of the catalysts. Roughness factor (R_f) was estimated from the ratio of double-layer capacitance (C_{dl}) between the working electrode and its corresponding smooth Ni electrode (assuming that the average double-layer capacitance of a smooth Ni electrode is 20 μF cm^{-2}), $R_f = C_{dl}/20$ μF cm^{-2} (Eq. S2). The C_{dl} was determined by measuring the capacitive current associated with double-layer charging from the scan-rate dependence of cyclic voltammetric stripping. A series of CV experiments at different scan rates e.g., 10, 20, 40, 60, 80, and 100 mV s^{-1} were performed in 0.5 M $KHCO_3$ with saturated carbon dioxide to calculate the C_{dl} of each catalyst. All experiments were performed using the same surface area.

The calculated number of active sites for each catalyst was obtained using the equation:

Density of active sites for catalyst (sites/cm^2) = Density of active sites for standard sample (sites/cm^2) × R_f (Eq. S3)

Additionally, the CO formation turn over frequency (TOF) of active sites for CO_2 reduction reaction in the Ni-NC_ATPA@CB and Ni-NC_TPA@CB was calculated at different overpotentials using the following equation:

CO formation TOF (s^{-1}) = i_0 ($A\ cm^{-2}$) × CO formation FE /([active sites density ($sites/cm^2$)] × [1.602×10^{-19} (C/e^-)] × [$2e^-/CO_2$]) (Eq. S4)

Faradaic efficiency (FE) measurement. The FE values of catalysts were calculated using $FE = \alpha nF/Q$ (Eq. S5), where α is the number of electrons transferred ($\alpha = 2$ for CO and H_2 production), n the number of moles for a given product, F Faraday's constant (96 485 C mol^{-1}), Q all the charge passed throughout the electrolysis process (measured by calculating the curve area of current density vs. time plot). CO and H_2 mole fractions of injected samples were calculated using GC calibration curve.

Computational details. Structure relaxation and total electronic energy calculations were performed using spin-polarized density functional theory (DFT) implemented in the Vienna ab initio simulation package (VASP)[1-3] with projector-augmented wave (PAW)[4] pseudopotential. We used PBE exchange-correlation functional[5] with the dispersion correction (D3).[6,7] A cut-off energy for the plane wave basis set was set to 400 eV.

Carbon coated Ni was modelled by a (3x3) graphene supercell and (3x3) atomic unit cell with three layers of Ni(111). The Ni(111) surface was constructed by a (3x3) atomic unit cell with four layers. The bottom two Ni layers were fixed to their optimized bulk positions. We used (5x5) graphene supercell containing 50 carbon atoms to construct pristine graphene, N-doped graphene and $Ni-N_4$ graphene ($Ni-N_4/Gr$). Graphene on Ni(111) and Ni(111) were calculated with the 3 x 3 x 1 Monkhorst-Pack mesh of k-points, while pristine graphene, N-doped graphene and $Ni-N_4/Gr$ were calculated with the 2 x 2 x 1.[8] All slab models included 18 Å of vacuum in the z-axis. The computational hydrogen electrode (CHE) model was employed to establish a free energy profile for electrochemical reactions.[9] We added -0.51 eV for a CO gas molecule to correct the inaccuracy of PBE with respect to the

experimental reaction enthalpies.[10] To account for the effect of solvent (water), we added an approximate solvation correction to *COOH and *CO by -0.25 eV and -0.1 eV, respectively.[10]

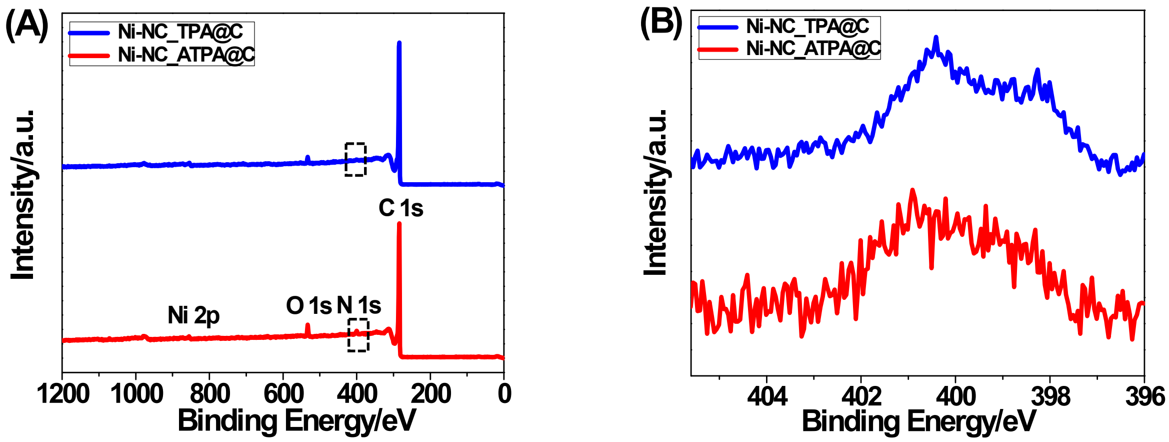

Fig. S1. (A) The wide-survey XPS spectra and (B) N 1s XPS spectra of Ni-NC_TPA@C and Ni-NC_ATPA@C.

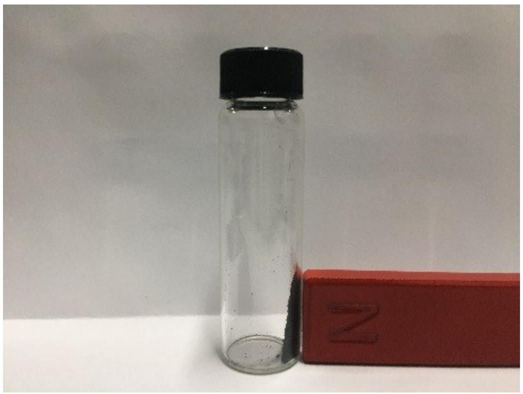

Fig. S2. Photograph of a vial with Ni-NC_ATPA@C in contact with a magnet, showing ferromagnetic properties of the sample.

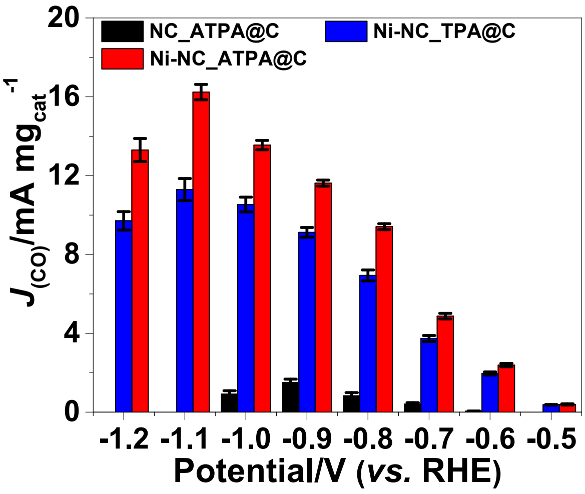

Fig. S3. CO partial current densities of Ni-NC_TPA@C, Ni-NC_ATPA@C, and NC_ATPA@C at various applied potentials based on the mass of total materials for the three catalysts.

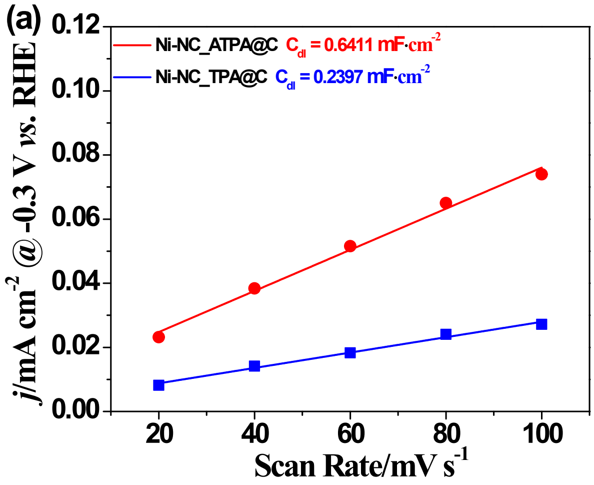

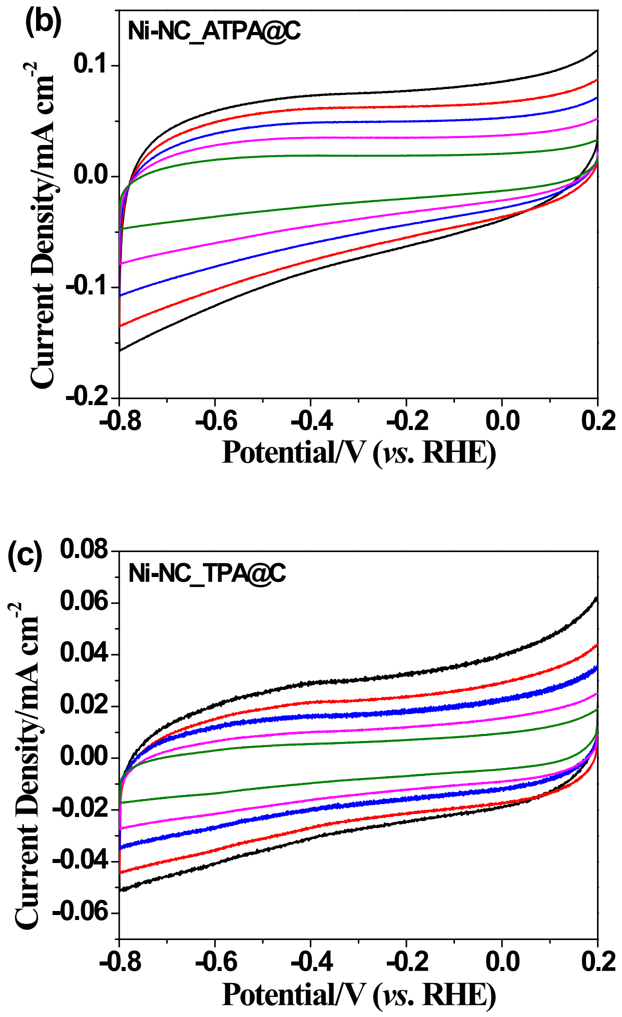

Fig. S4. (a) Current density of CV experiments at -0.3 V (*vs*. RHE) as a function of scan rate. The slope of this line shows double-layer capacitor for each catalyst. (b), (c) Scan-rate dependence of cyclic voltammetric stripping. A series of CV experiments at different scan rates e.g., 20, 40, 60, 80, and 100 mV s^{-1} were performed in 0.1 M $KHCO_3$ to calculate the C_{dl} of Ni-NC_ATPA@C and Ni-NC_TPA@C.

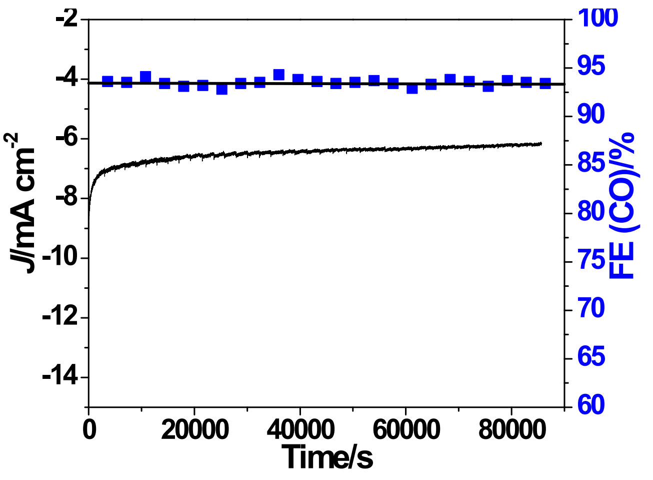

Fig. S5. Current–time response, along with CO FE, of Ni-NC_ATPA@C on glassy carbon electrode for CO_2 reduction at -0.7 V (*vs*. RHE) in CO_2-saturated 0.5 M aqueous $KHCO_3$.

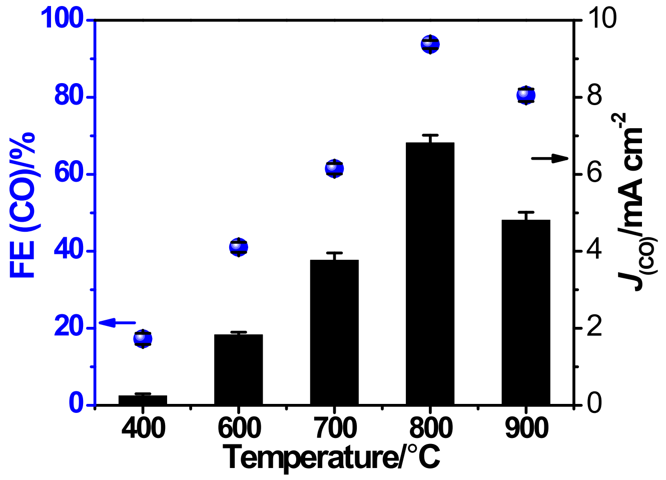

Fig. S6. CO FEs and partial current densities at -0.7 V (*vs*. RHE) of Ni-NC_ATPA@C obtained at 400, 600, 700, 800, and 900 ºC in Ar for 2 h.

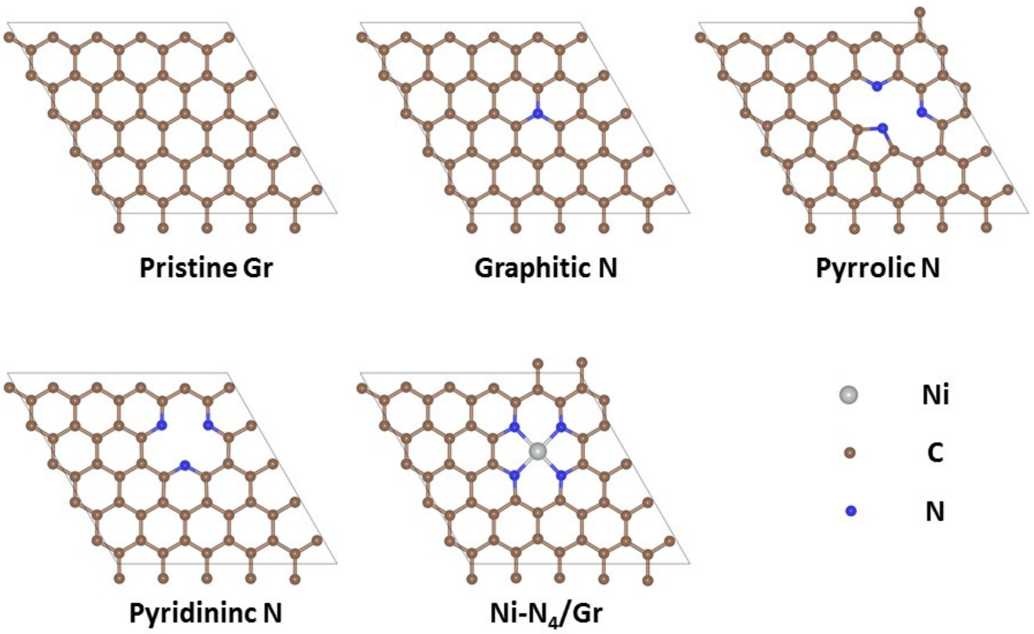

Fig. S7. Calculation models for pristine graphene, N-doped graphene, and Ni-N_4/Gr.

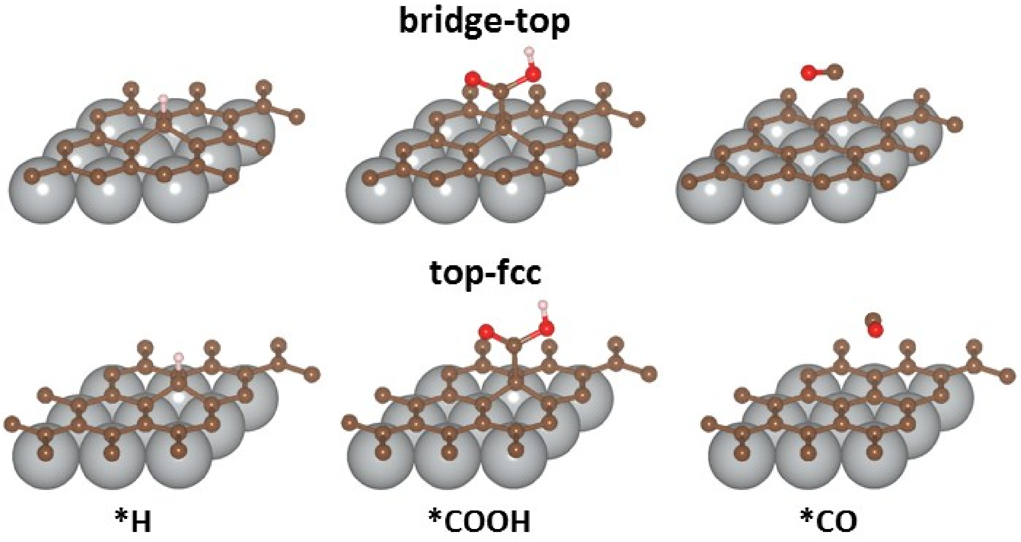

Fig. S8. Optimized geometries of reaction intermediates for ECR and HER on Gr/Ni(111). Bottom two Ni layers were omitted for clarity. CO is not chemically adsorbed on Gr/Ni(111).

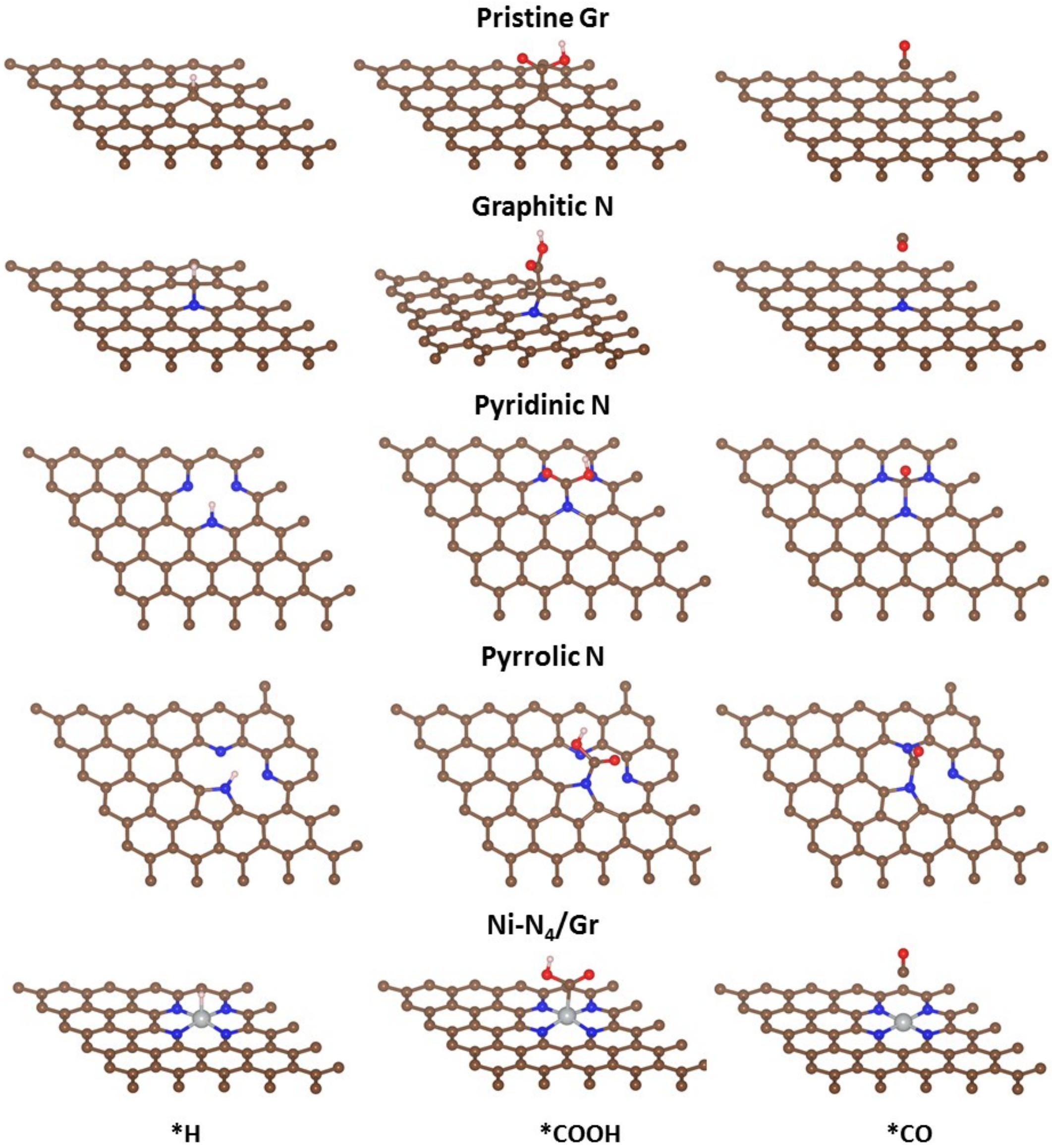

Fig. S9. Optimized geomietries of reaction intermediates for ECR and HER on pristine graphene, N-doped graphene and Ni-N_4/Gr. CO is not chemically adsorbed on pristine Gr, graphitic N and Ni-N_4/Gr. For graphitic N, adjacent C atom is more active than N atom.

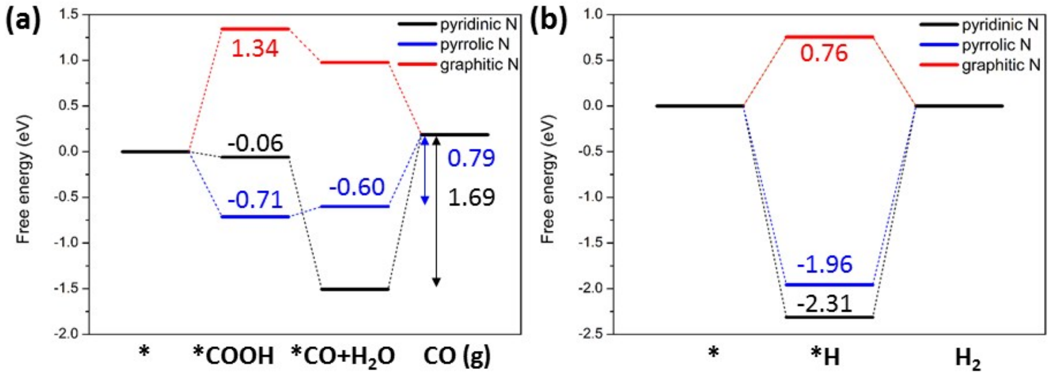

Fig. S10. Free energy diagrams for (a) ECR and (b) HER on N-doped graphene.

Table S1. Summary of Ni catalysts reported for electrocatalytic CO_2 reduction

Ni catalysts	Electrolytes	CO partial current density	Onset potential (*vs.* RHE)	CO FE	Reference
Single Ni sites					
C-Zn_1Ni_4 ZIF-8	1 M $KHCO_3$	71.5±2.9 mA cm^{-2} @-1.03 V (*vs.* RHE)	-0.43 V	98% @-0.63 V (*vs.* RHE)	11
Ni SAs/N-C	0.5 M $KHCO_3$	7.37 mA cm^{-2} @-1.0 V (*vs.* RHE)	-0.57 V	71.9% @-0.9 V (*vs.* RHE)	12
Ni-N-C	0.1 M $KHCO_3$	8.2 mA cm^{-2} @-0.64 V (*vs.* RHE)	N/A	96% @-0.64 V (*vs.* RHE)	13
Ni-NG	0.5 M $KHCO_3$	11 mA cm^{-2} @-0.64 V (*vs.* RHE)	-0.31 V	95% @-0.49 V (*vs.* RHE)	14
Ni-N-C	0.5 M $KHCO_3$	3.6 mA cm^{-2} @-0.67 V (*vs.* RHE)	-0.47 V	93% @-0.67 V (*vs.* RHE)	15
Ni-N-C	0.5 M $KHCO_3$	28.6 mA cm^{-2} @-0.81 V (*vs.* RHE)	-0.4 V	99% @-0.81 V (*vs.* RHE)	16
Ni-CTF	0.1 M $KHCO_3$	2.45 mA cm^{-2} @-1.1 V (*vs.* RHE)	-0.5 V	90% @-0.8 V (*vs.* RHE)	17
NiSA-N-CNTs	0.5 M $KHCO_3$	23.5 mA cm^{-2} @-0.7 V (*vs.* RHE)	-0.31 V	91.3% @-0.7 V (*vs.* RHE)	18
Ni^{2+}@NG	0.5 M $KHCO_3$	24 mA cm^{-2} @-0.88 V (*vs.* RHE)	-0.38 V	92% @-0.68 V (*vs.* RHE)	19
Ni-N-C	0.1 M $KHCO_3$	10 mA cm^{-2} @-0.8 V (*vs.* RHE)	-0.38 V	85% @-0.78 V (*vs.* RHE)	19
A-Ni-NG	0.5 M $KHCO_3$	22 mA cm^{-2} @-0.7 V (*vs.* RHE)	-0.3 V	97% @-0.61 V (*vs.* RHE)	20
Ni-N-Gr	0.1 M $KHCO_3$	0.2 mA cm^{-2} @-0.65 V (*vs.* RHE)	-0.5 V	95% @-0.7 V (*vs.* RHE)	21
Ni nanoparticle catalysts					

Ni NPs/N-C	0.5 M $KHCO_3$	~0.42 mA cm^{-2} @-1.0 V (*vs*. RHE)	N/A	~24% @-0.9 V (*vs*. RHE)	12
Ni-CNTs	0.5 M $KHCO_3$	~0.25 mA cm^{-2} @-0.7 V (*vs*. RHE)	N/A	~5% @-0.55 V (*vs*. RHE)	18

References

1. G. Kresse and J. Furthmüller, *Phys. Rev. B*, 1996, **54**, 11169-11186.
2. G. Kresse and J. Furthmüller, *Comput. Mater. Sci.*, 1996, **6**, 15-50.
3. G. Kresse and D. Joubert, *Phys. Rev. B*, 1999, **59**, 1758-1775.
4. P. E. Blöchl, *Phys. Rev. B*, 1994, **50**, 17953-17979.
5. J. P. Perdew, K. Burke and M. Ernzerhof, *Phys. Rev. Lett.*, 1996, **77**, 3865-3868.
6. S. Grimme, J. Antony, S. Ehrlich and H. Krieg, *J. Chem. Phys.*, 2010, **132**, 154104-154119.
7. S. Grimme, S. Ehrlich and L. Goerigk, *J. Comput. Chem.*, 2011, **32**, 1456-1465.
8. H. J. Monkhorst and J. D. Pack, *Phys. Rev. B*, 1976, **13**, 5188-5192.
9. J. K. Nørskov, J. Rossmeisl, A. Logadottir, L. Lindqvist, J. R. Kitchin, T. Bligaard and H. Jonsson, *J. Phys. Chem. B*, 2004, **108**, 17886-17892.
10. A. A. Peterson, F. Abild-Pedersen, F. Studt, J. Rossmeisl and J. K. Nørskov, *Energy Environ. Sci.*, 2010, **3**, 1311-1315.
11. C. C. Yan, H. B. Li, Y. F. Ye, H. H. Wu, F. Cai, R. Si, J. P. Xiao, S. Miao, S.H. Xie, F. Yang, Y.S. Li, G.X. Wang and X. H. Bao, *Energy Environ. Sci.*, 2018, **11**, 1204-1210.
12. C. M. Zhao, X. Y. Dai, T. Yao, W. X. Chen, X. Q. Wang, J. Wang, J. Yang, S. Q. Wei, Y. Wu and Y. D. Li, *J. Am. Chem. Soc.*, 2017, **139**, 8078-8081.
13. F. P. Pan, W. Deng, C. Justiniano and Y. Li, *Appl Catal. B*, 2018, **226**, 463-472.
14. K. Jiang, S. Siahrostami, T. T. Zheng, Y. S. Hu, S. Hwang, E. Stavitski, Y. Peng, J. Dynes, M. Gangisetty, D. Su, K. Attenkofer and H.T. Wang, *Energy Environ. Sci.*, 2018, **11**, 893-903.

15. X. M. Hu, H. H. Hval, E. T. Bjerglund, K. J. Dalgaard, M. R. Madsen, M. M. Pohl, E. Welter, P. Lamagni, K. B. Buhl, M. Bremholm, M. Beller, S. U. Pedersen, T. Skrydstrup and K. Daasbjerg, *ACS Catal.*, 2018, **8**, 6255-6264.
16. X. G. Li, W. T. Bi, M. L. Chen, Y. X. Sun, H. X. Ju, W. S. Yan, J. F. Zhu, X. J. Wu, W. S. Chu, C. Z. Wu and Y. Xie , *J. Am. Chem. Soc.* 2017, **139**, 14889-14892.
17. P. Su, K. Lwase, T. Harada, K. Kamiya and S. Nakanishi, *Chem. Sci.* 2018, **9**, 3941-3947.
18. Y. Cheng, S. Y. Zhao, B. Johannessen, J. P. Veder, M. Saunders, M. R. Rowles, M. Cheng, C. Liu, M. F. Chisholm, R. De Marco, H. M. Cheng, S. Z. Yang and S. P. Jiang, *Adv. Mater*. 2018, **30**, 1706287.
19. W. T. Bi, X. G. Li, R. You, M. L. Chen, R. L. Yuan, W. X. Huang, X. J. Wu, W. S. Chu, C. Z. Wu and Y. Xie, *Adv. Mater.* 2018, **30**, 1706617.
20. W. Ju, A. Bagger, G. P. Hao, A. S. Varela, I. Sinev, V. Bon, B. Roldan Cuenya, S. Kaskel, J. Rossmeisl and P. Strasser, *Nat. Commun*. 2017, **8**, 944-953.
21. H. B. Yang, S. F. Hung, S. Liu, K. D. Yuan, S. Miao, L. P. Zhang, X. Huang, H. Y. Wang, W. Z. Cai, R. Chen, J. J. Gao, X. F. Yang, W. Chen, Y. Q. Huang, H. M. Chen, C. M. Li, T. Zhang and B. Liu, *Nat. Energy,* 2018, **3**, 140-147.
